# Short-term cardiac outcome in survivors of COVID-19: a systematic study after hospital discharge

**DOI:** 10.1007/s00392-020-01800-z

**Published:** 2021-01-22

**Authors:** Leonardo A. Sechi, Gianluca Colussi, Luca Bulfone, Gabriele Brosolo, Andrea Da Porto, Maddalena Peghin, Vincenzo Patruno, Carlo Tascini, Cristiana Catena

**Affiliations:** 1 Division of Internal Medicine (L.A.S., L.B., A.D.P.), Department of Medicine, University of Udine, 33100 Udine, Italy; 2 Department of Medicine, Cardiovascular Unit (G.C., G.B., C.C.), University of Udine, 33100 Udine, Italy; 3 Division of Infectious Diseases (M.P., C.T.), Department of Medicine, University of Udine, 33100 Udine, Italy; 4 Division of Pneumology (V.P.), Department of Medicine, University of Udine, 33100 Udine, Italy; 5 Department of Medicine, Clinica Medica, University of Udine, University Hospital, Building 8, 33100 Udine, Italy

**Keywords:** COVID-19, Diastolic function, Echocardiography, Lung ultrasound, Systolic function, Tissue-Doppler imaging

## Abstract

**Background:**

COVID-19 has caused considerable morbidity and mortality worldwide and cardiac involvement has been reported during infection. The short-term cardiac outcome in survivors of COVID-19 is not known.

**Objective:**

To examine the heart of patients who survived COVID-19 and to compare the cardiac outcome between patients who recovered from mild-to-moderate or severe illness.

**Methods:**

With use of ECG and echocardiography, we examined the heart of 105 patients who had been hospitalized with COVID-19 and were consecutively recruited after hospital discharge while attending follow-up visits. Survivors of COVID-19 were compared with 105 matched controls. We also compared the cardiac outcome and lung ultrasound scan between COVID-19 patients who had mild-to-moderate or severe illness.

**Results:**

Cardiac data were collected a median of 41 days from the first detection of COVID-19. Symptoms were present in a low percentage of patients. In comparison with matched controls, no considerable structural or functional differences were observed in the heart of survivors of COVID-19. Lung ultrasound scan detected significantly greater residual pulmonary involvement in COVID-19 patients who had recovered from severe than mild-to-moderate illness. No significant differences were detected in ECG tracings nor were found in the left and right ventricular function of patients who had recovered from mild-to-moderate or severe illness.

**Conclusions:**

In a short-term follow-up, no abnormalities were identified in the heart of survivors of COVID-19, nor cardiac differences were detected between patients who had different severity of illness. With the limitations of a cross-sectional study, these findings suggest that patients who recover from COVID-19 do not have considerable cardiac sequelae.

**Graphic abstract:**

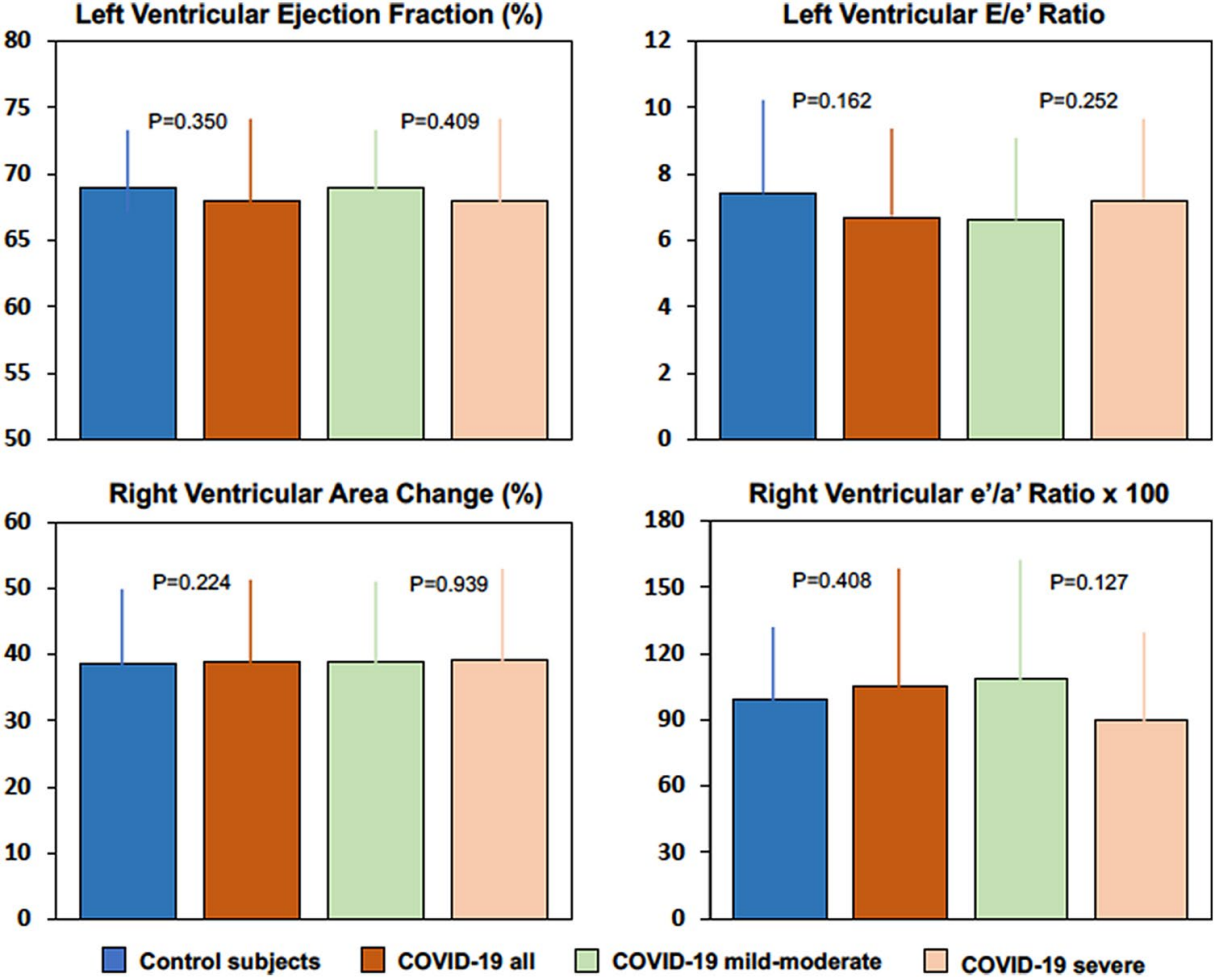

**Supplementary Information:**

The online version contains supplementary material available at 10.1007/s00392-020-01800-z.

## Introduction

The dreadful pandemic caused by Severe Acute Respiratory Syndrome-Coronavirus-2 (SARS-CoV-2) represents an unprecedented challenge for the healthcare community and has already left behind thousands of victims worldwide. High infectivity and ability to be transmitted even by asymptomatic subjects have resulted in a rapid spread of infection throughout all continents, causing a disease that has been named coronavirus disease 2019 (COVID-19) [1]. COVID-19 involves primarily the respiratory system, presenting with a broad range of clinical pictures, from a very mild flulike illness to fatal acute respiratory distress syndrome (ARDS) [2]. Notably, additional cardiac, vascular, gastrointestinal, renal, hepatic, and neurologic involvement has been reported in COVID-19 patients [3], supporting the tenet of a systemic spread of the virus.

The COVID-19 outbreak has triggered an explosion of clinical and experimental research which has already provided important insights into the pathogenesis of this infection and its complications. Many reports from geographical areas that have endured the heaviest burden of deaths caused by COVID-19 have provided evidence that older age and preexisting cardiovascular conditions enhance vulnerability of patients to infection [4]. On the other hand, studies prevalently conducted in China where the outbreak originated, indicated cardiac injury as a prominent feature complicating the course of COVID-19 [5]. Cardiac involvement was in fact demonstrated in a substantial proportion of patients with COVID-19 by detection of increased levels of biochemical markers such as troponins and brain-type natriuretic peptide and was associated with elevated mortality [6, 7]. Mechanisms of cardiac injury in COVID-19 are not clear, but might involve direct cardiac localization of virus, myocardial stress due to hypoxemia caused by respiratory failure, indirect injury linked to systemic inflammatory response causing an uncontrollable cytokine storm, or a combination of them [8].

Despite high infectivity, SARS-CoV-2 virulence is mild with a relatively low fatality rate [9, 10]. Consequently, the majority of patients survive COVID-19 leaving open an important question about the possible organ sequelae of infection. Because the heart has been reported to be critically involved in COVID-19, we undertook a study to investigate the short-term cardiac outcome of patients who recovered from disease. By the use of cardiac ultrasound, we examined the structure and function of the heart of patients who had been hospitalized with COVID-19 and compared the cardiac characteristics of patients who recovered from mild-to-moderate or severe illness. We also examined the association of cardiac characteristics with biochemical markers that were measured during the acute phase of infection.

## Methods

### Study design, setting, and participants

This observational study was conducted at the University Hospital-University of Udine, a quaternary acute care regional hospital serving an area of more than 700,000 people in northeast of Italy. We examined the cardiac structure and function by echocardiography in consecutive COVID-19 patients that attended the dedicated outpatient Clinic after hospital discharge, from April 15 to May 15, 2020. From March 15 to April 15, 2020, patients had presented with fever and/or respiratory symptoms at specific COVID-19 points of care that were set up in the Infectious Disease and the Emergency Units of the Hospital and had positive test results for the SARS-CoV-2 on a nasopharyngeal swab. Tests were conducted with a reverse-transcriptase-polymerase-chain-reaction (RT-PCR) by the Central Laboratory of the Hospital that serves as a facility for SARS-CoV-2 detection in the whole area and COVID-19 was diagnosed on the basis of the World Health Organization guidance [11]. All patients with a positive test result for SARS-CoV-2 were assessed according to a clinical severity score (Supplementary Table 1). The score was specifically developed for the COVID-19 outbreak by the Italian Society of Emergency Medicine by setting five levels [12] that incorporate all the criteria that are currently used to define severity of illness [9]. Depending upon the severity score, patients were admitted either to a COVID-19 isolation unit for close monitoring of clinical progression and oxygen support with high-flow nasal cannula (*level 1* and *level 2*), a COVID-19 isolation sub-intensive care unit for oxygen support with continuous positive airway pressure (*level 3*), or a COVID-19 isolation intensive care unit for invasive mechanical ventilation with orotracheal intubation (*level 4* and *level 5*). The severity score was reassessed after admission, and the highest level reached during hospitalization was considered for analysis. Patients within levels 1–2 were assigned to a mild-to-moderate COVID-19 group and patients within levels 3–5 to a severe COVID-19 group. Survivors of COVID-19 were discharged from the Hospital with scheduled follow-up visits, during which clinical variables were reassessed, nasopharyngeal swab was repeated, blood samples for laboratory tests were collected, and ECG, lung ultrasound, and echocardiography were performed. Patients who attended follow-up visits during that period were 61% of those who were discharged from March 15 to April 15, 2020, and therefore a selection bias cannot be excluded. The study complied with the Declaration of Helsinki and received approval from the Internal Review Board of the Department of Medicine under a fast-track review process. All patients gave their written informed consent to the study.

### Data collection

Patients’ data were collected from the University Hospital electronic database. The database is continuously and timely updated and contains all the data of patients registered to the Hospital, including patients’ demographic, anthropometric, and clinical variables, laboratory test results, historical and current diagnoses together with history of previous hospitalizations, and historical and current medication list.

### Control subjects

Cardiac ultrasound variables that were measured after hospital discharge in survivors of COVID-19 were compared with those of a control group of COVID-19 negative individuals. These individuals were selected by use of a nearest neighbor matching strategy from an internal database of subjects that are representative of the general population of the same geographical area of COVID-19 patients. The database contains demographic, anthropometric, and clinical data, laboratory test results, and cardiac ultrasound measurements of more than 1000 subjects who had consecutively attended the outpatient service of the Internal Medicine and Cardiovascular Clinic of the University Hospital from January 2018 to December 2019. Exact matching was used to adjust for age, sex, and hypertension prevalence.

### Echocardiography

We performed two-dimensional, Doppler, and tissue-Doppler imaging (TDI) transthoracic echocardiography in survivors of COVID-19 after hospital discharge. Examination was performed with use of appropriate personal protective equipment [13] by experienced investigators who were blinded to the clinical and laboratory data, in accordance with the joint recommendations of the American Society of Echocardiography and the European Association of Cardiovascular Imaging [14], as described previously [15] (Supplementary methods). Measurements of left and right atrial volumes, left and right ventricular dimensions, left ventricular mass and geometry, and systolic pulmonary artery pressure were obtained by use of two-dimensional and Doppler ultrasound. Left and right ventricular systolic and diastolic function were assessed by use of two-dimensional, Doppler, and TDI echocardiography.

### Lung ultrasound

Lung ultrasound was performed by scanning two different areas in the anterior, lateral, and posterior region of each lung. A point score was obtained for each examined area as follows [16]: 0 points for A lines (normal visualization of the pleural lines with lung sliding that indicate normal lung aeration); 1 point for B lines (shining lines perpendicular to the pleural space erasing A lines at the edge of the screen due to a reverberation artifact through edematous interlobular septa) that indicate moderate loss of lung aeration; 2 points for lung consolidation. Therefore, for each patient the lung ultrasound score was between 0 and 24 points.

### Statistical analysis

Normality of variables distribution was assessed with the Shapiro–Wilk test. Normally distributed continuous variables are reported as mean ± standard deviation (SD) and skewed variables as median ± interquartile range (IQR). Independent sample Student’s *t* test was used for comparison between groups of normally distributed variables and the Mann–Whitney *U* test for comparison of skewed variables. Point differences between means or medians with 95% confidence intervals are reported together with *P* values. Categorical variables are reported as counts, and percentages and differences are reported as percentage point with 95% confidence intervals together with *P* values that were tested by use of the Fisher’s exact test. The primary analysis was an evaluation of cardiac ultrasound characteristics of patients who survived COVID-19 in a comparison with a control group of matched subjects and of COVID-19 patients who had recovered from mild-to-moderate or severe illness. Analyses were conducted using Stata software, version 12.1 (StataCorp).

## Results

### Study patients and control subjects

From April 15 to May 15, 2020, 105 patients who were discharged from the hospital attended scheduled follow-up visits with ECG registration and heart and lung ultrasound examination. The date of COVID-19 diagnosis was considered as the index date to calculate the timing of cardiac ultrasound examination that was performed after a median of 41 days (IQR: 37–44 days). After hospital discharge, lymphocyte and platelets counts increased significantly (both *P* < 0.001) whereas serum levels of creatine kinase, lactate dehydrogenase, C-reactive protein, procalcitonin, and D-dimer decreased (all *P* < 0.001) (Supplementary Table 2). At follow-up, 25 (24%) of 105 COVID-19 patients still had positive test results for SARS-CoV-2 on the nasopharyngeal swab.

The demographic and clinical characteristics of COVID-19 survivors and controls are shown in Supplementary Table 3. Body mass index and blood pressure were appropriately balanced in the two groups. A history of hypertension was present in 30% of study patients and controls and current smokers were 15% and 16%, respectively. All the additional coexisting conditions had a prevalence of less than 10% in patients and controls. Table 1 summarizes the echocardiographic variables of COVID-19 patients and controls. Dimensions of the left and right heart chambers were comparable in COVID-19 survivors and controls and also left ventricular mass and relative wall thickness did not differ between the two groups. Left ventricular ejection fraction and fractional shortening, mitral annular plane systolic excursion (MAPSE) and TDI peak systolic velocity (S’) values did not differ between COVID-19 survivors and controls. All markers of left ventricular diastolic function obtained by Doppler ultrasound (peak *E* velocity; *E*/*A* ratio; *E* wave deceleration time; tricuspid regurgitation peak velocity) and TDI (mitral annular *e*’ velocity; *e*’/*a*’ ratio; *E*/*e*’ ratio) were comparable between COVID-19 survivors and controls. Variables of right ventricular systolic (right ventricular fractional shortening; tricuspid annular plane systolic excursion; tricuspid annular peak systolic velocity) and diastolic (tricuspid annular *e*’ velocity and *e*’/*a*’ ratio) function also had comparable values in COVID-19 survivors and controls. The systolic pulmonary artery pressure was 22 mm Hg in both the study patients and controls. High-sensitivity serum troponin was measured at the time of echocardiographic assessment and was below the upper limit of normal values according to local hospital criteria as defined by the 99th percentile of healthy reference population (males 79 ng/l; females 54 ng/ml) in all patients (median 5 ng/l; IQR 1–13).

**Table 1:** Cardiac ultrasound characteristics of survivors of COVID-19 and matched controls

Characteristic	Study patients (*N* = 105)	Controls (*N* = 105)	Difference (95% CI)	*P* value
Left heart				
Left atrial volume—ml	42 (19)	40 (11)	2 (-1 to 6)	0.136
Left ventricular end-diastolic diameter—mm	47 (6)	49 (5)	-2 (-3 to 1)	0.060
Left ventricular end-systolic diameter—mm	28 (6)	29 (6)	− 1 (− 2 to 1)	0.158
Interventricular septum—mm	9.1 (1.9)	9.0 (1.8)	0.1 (− 0.2 to 0.9)	0.557
Posterior wall—mm	9.0 (1.8)	8.9 (1.6)	0.1 (− 0.3 to 0.9)	0.168
Left ventricular mass index—g/m^2.7^	34.6 (10.7)	35.5 (10.6)	− 0.9 (− 4.4 to 2.2)	0.572
Relative wall thickness—%	0.386 (0.098)	0.369 (0.072)	0.017 (− 0.009 to 0.060)	0.117
Left ventricular ejection fraction—%	68 (10)	69 (7)	− 1 (− 4 to 1)	0.350
Left ventricular fractional shortening—%	39.1 (7.1)	40.5 (9.8)	− 1.4 (− 4.0 to 1.2)	0.293
Mitral annular plane systolic excursion—mm	13.9 (2.4)	15.0 (2.6)	− 1.1 (− 2.9 to 0.4)	0.264
Mitral annular peak systolic velocity *S*’—cm/s^a^	9.2 (2.1)	8.2 (2.0)	1.0 (− 0.5 to 1.6)	0.097
Peak *E* wave velocity—cm/s	74 (21)	71 (20)	3 (− 4 to 9)	0.822
*E*/*A* ratio^b^	1.24 (0.56)	1.27 (0.39)	− 0.03 (− 0.22 to 0.07)	0.316
E wave deceleration time—ms	208 (54)	198 (49)	10 (− 5 to 26)	0.180
Mitral annular *e*′ velocity—cm/s^a^	11.2 (3.6)	10.3 (3.2)	0.9 (− 1.1 to 1.9)	0.180
Mitral annular *e*′/*a*′ ratio^a^	1.05 (0.56)	1.12 (0.47)	− 0.07 (− 2.20 to 0.14)	0.424
Mitral *E*/*e*′ ratio^a^	6.68 (2.29)	7.42 (3.04)	− 0.74 (− 1.53 to 0.04)	0.162
Right heart				
Right atrial volume—ml	42 (20)	41 (13)	1 (− 3 to 7)	0.192
Right ventricular diastolic area—cm^2^	21.8 (4.9)	20.7 (5.3)	1.1 (− 0.4 to 2.6)	0.154
Right ventricular systolic area—cm^2^	13.2 (3.9)	12.7 (3.8)	0.5 (− 1.0 to 3.6)	0.167
Right ventricular fractional area change—%	0.389 (0.125)	0.386 (0.116)	0.030 (− 0.079 to 0.110)	0.224
Tricuspid annular plane systolic excursion—mm	21.4 (4.0)	22.6 (4.2)	− 1.2 (− 2.4 to 0.05)	0.161
Tricuspid annular peak systolic velocity *S*′—cm/s^a^	13.7 (3.0)	13.5 (2.8)	0.2 (− 0.6 to 1.1)	0.570
Tricuspid regurgitation peak velocity—m/s^c^	2.28 (0.51)	2.26 (0.50)	0.02 (− 0.24 to 0.28)	0.881
Tricuspid annular e′ velocity—cm/s^a^	14.3 (3.7)	13.6 (3.1)	0.7 (0.4 to 1.7)	0.213
Tricuspid annular *e*’/*a*′ ratio^a^	1.05 (0.54)	0.99 (0.33)	0.06 (− 0.08 to 0.20)	0.408
Systolic pulmonary artery pressure—mm Hg^c^	22 (8)	22 (6)	0 (− 4 to 4)	0.899

Values are means (SD). The 95% confidence intervals (CI) have not been adjusted for multiple testing and should not be used to infer definitive effects. Cases of COVID-19 were diagnosed between March 15 and April 15, 2020. Controls were selected by use of a nearest neighbor matching strategy from a large internal database of subjects that are representative of the general population and were exactly matched for age, sex, and hypertension, whereas propensity score models were used for matching of body mass index, smoking, diabetes mellitus, hyperlipidemia, coronary artery disease, congestive heart failure, atrial fibrillation, and chronic obstructive pulmonary disease

^a^Tissue-Doppler imaging variables. *S*’, peak systolic velocities were measured either at the mitral or tricuspid annulus with the Doppler beam directed parallel to the myocardial walls; *e*’, early-diastolic myocardial velocity was measured either at the mitral or tricuspid annulus; *a*’, late-diastolic myocardial velocity was measured either at the mitral or tricuspid annulus

^b^*E*, early-wave trans-mitral peak velocity; *A*, late-wave trans-mitral peak velocity

^c^Measurement of tricuspid regurgitation peak velocity and calculation of systolic arterial pulmonary pressure could be performed in in 84.8% of study patients and 80.0% of controls, and the mean value was imputed in the remaining subjects

### Patients with mild-to-moderate or severe COVID-19

Of 105 study patients, 27 (26%) had been hospitalized with severe COVID-19 requiring mechanical ventilation with either continuous positive airway pressure or orotracheal intubation and the remaining 78 (74%) with mild-to-moderate disease. Table 2 shows the clinical, and laboratory that were measured in the COVID-19 patients during hospitalization. Male sex was more frequent (*P* = 0.012) in patients with severe (74%) than mild-to-moderate (46%) COVID-19. Patients with severe COVID-19 had lower lymphocyte count and higher median values of transaminases, lactate dehydrogenase, C-reactive protein, procalcitonin, D-dimer, and interleukin-6 than patients with mild-to-moderate COVID-19 (all *P* < 0.001). Drug treatments that were used in COVID-19 patients during hospitalization are shown in Supplementary Table 4. Hydroxychlorochine (63% and 96%, respectively) and azithromycin (39% and 37%, respectively) were used most frequently in both mild-to-moderate and severe COVID-19. The frequency of use of antiviral agents such as darunavir–cobicistat and lopinavir/ritonavir in patients with severe COVID-19 was twofold of patients with mild-to-moderate COVID-19. Moreover, tocilizumab, an interleukin-6 antagonist, was used in a substantial proportion (56%) of patients with severe COVID-19. At follow-up, symptoms were present in a limited percentage of patients (Table 3) with no differences between those who recovered from mild-to-moderate or severe illness. ECG tracings showed also no significant differences between COVID-19 patients who had mild-to-moderate or severe illness. As shown in Table 4, left and right atrial and ventricular dimensions, left ventricular mass and relative wall thickness, markers of left and right ventricular systolic and diastolic function, and systolic pulmonary arterial pressure were comparable in patients who recovered from mild-to-moderate or severe COVID-19. Lung ultrasound examination detected mild signs of residual pulmonary involvement that were significantly greater in patients who had been hospitalized with severe COVID-19 than those with mild-to-moderate disease.

**Table 2:** Clinical characteristics and laboratory findings of COVID-19 patients with mild-to-moderate and severe disease during hospitalization

Characteristic	Mild-to-moderate COVID-19 (*N* = 78)	Severe COVID-19 (*N* = 27)	Difference (95% CI)	*P* value
Clinical characteristics				
Age—years	56.7 (15.1)	59.7 (11.9)	− 3.0 (− 9.4 to 3.3)	0.348
Female sex—no. (%)	42 (54)	7 (26)	28 (6 to 50)	0.012
Body mass index—kg/m^2^	25.80 (4.97)	27.43 (3.96)	− 1.63 (− 3.73 to 0.46)	0.125
Systolic blood pressure—mm Hg	134 (19)	135 (17)	− 1.1 (− 10.4 to 8.3)	0.821
Diastolic blood pressure—mm Hg	78 (11)	81 (12)	− 2.9 (− 8.5 to 2.7)	0.305
Current smokers—no. (%)	14 (18)	2 (7)	12 (− 2 to 18)	0.231
Hypertension—no. (%)	20 (26)	11 (41)	− 15 (− 35 to 5)	0.149
Laboratory findings				
Hemoglobin—g/dl	14.0 (1.2)	14.0 (1.4)	0.0 (− 5.8 to 5.6)	0.971
White cell count—per mm^3^	5812 (2786)	6392 (3442)	− 580 (− 1,963 to 803)	0.407
Lymphocyte count—per mm^3^	1041 (418)	748 (326)	275 (88 to 462)	< 0.001
Platelet count—per mm^3^	201,000 (80,000)	208,000 (67,000)	− 7000 (− 42,110 to 28,130)	0.396
Glucose—mmol/liter	6.22 (1.50)	6.83 (2.22)	− 0.61 (− 1.41 to 0.23)	0.155
Creatinine—mmol/liter	85.8 (24.8)	89.3 (23.9)	− 3.5 (− 14.9 to 7.8)	0.532
Glomerular filtration rate—ml/min	78 (21)	78 (17)	0.0 (− 8.9 to 9.2)	0.973
Sodium—mmol/liter	139 (3)	138 (4)	1.0 (− 0.5 to 2.2)	0.232
Potassium—mmol/liter	3.89 (0.42)	3.87 (0.52)	0.02 (− 0.20 to 0.23)	0.924
Alanine aminotransferase—U/liter	20 [13–28]	28 [18–43]	− 8 (− 15 to − 2)	0.005
Aspartate aminotransferase—U/liter	24 [19–31]	34 [25–46]	− 10 (− 15 to − 3)	0.003
Total bilirubin—mmol/liter	8.21 [7.01–10.52]	8.21 [6.41–11.97]	0.0 (− 1.20 to 2.00)	0.673
Creatine kinase—U/liter	81 [60–118]	118 [69–186]	− 37.0 (− 71.0 to 3.0)	0.083
Lactate dehydrogenase—U/liter	422 [345–596]	694 [475–870]	− 272 (− 330 to − 101)	< 0.001
C-reactive protein—mg/liter	37.2 [7.1–80.5]	106.1 [49.0–168.5]	− 68.9 (− 97.2 to − 20.9)	< 0.001
Procalcitonin—ng/ml	0.05 [0.03–0.10]	0.14 [0.06–0.39]	− 0.9 (− 1.4 to − 0.3)	< 0.001
D-dimer—ng/ml	424 [287–757]	1102 [548–2166]	− 678 (− 1061 to − 268)	< 0.001
B-type natriuretic peptide—pg/ml	21 [6–47]	25 [14–44]	− 4.0 (− 14–0 to 7.0)	0.424
Interleukin 6—pg/ml^a^	18 [8–27]	47 n	− 35 (− 62 to − 22)	< 0.001

Normally distributed values are shown as means (SD). Variables with skewed distribution are shown as medians [interquartile range]. The 95% confidence intervals (CI) have not been adjusted for multiple testing and should not be used to infer definitive effects. To convert the values for glucose to milligrams per deciliter, divide by 0.0555; to convert the values for creatinine to milligrams per deciliter, divide by 88.434; to convert the values for total bilirubin to milligrams per deciliter, divide by 17.104. Glomerular filtration rate was estimated by the Modification of Diet in Renal disease (MDRD) formula

^a^Measurement of serum interleukin 6 was done in 80.8% of patients with mild-to-moderate COVID-19 and 92.6% of patients with severe COVID-19, and the mean value was imputed in the remaining patients

**Table 3:** Clinical and electrocardiographic characteristics of COVID-19 patients with mild-to-moderate and severe disease at follow-up

Characteristic	Mild-to-moderate COVID-19 (*N* = 78)	Severe COVID-19 (*N* = 27)
Fever (> 37.5 °C)—no. (%)	3 (4)	1 (4)
Dyspnea—no. (%)	4 (5)	1 (4)
Cough—no. (%)	3 (4)	1 (4)
Chest pain—no. (%)	3 (4)	2 (7)
Fatigue—no. (%)	5 (6)	2 (7)
Gastrointestinal—no. (%)	2 (3)	0 (0)
Neurological—no. (%)	5 (6)	1 (4)
Dysgeusia/anosmia—%	7 (9)	2 (7)
Normal sinus rhythm—no. (%)	72 (92)	24 (89)
Atrial fibrillation/flutter—no. (%)	6 (8)	3 (11)
PQ-segment > 0.20 s—no. (%)	6 (8)	2 (7)
Right bundle-branch block—no. (%)	6 (8)	3 (11)
Left bundle-branch block—no. (%)	1 (1)	0 (0)
ST-segment elevation/depression—no. (%)	5 (6)	1 (4)
T-wave abnormalities—no. (%)	10 (13)	4 (15)
Long QTc—no. (%)	1 (1)	0 (0)

**Table 4:** Cardiac and lung ultrasound characteristics of COVID-19 patients with mild-to-moderate and severe disease at follow-up

Characteristic	Mild-to-moderate COVID-19 (*N* = 78)	Severe COVID-19 (*N* = 27)	Difference (95% CI)	*P* value
Left heart				
Left atrial volume—ml	42 (20)	42 (17)	− 0.0 (− 9 to 8)	0.914
Left ventricular end-diastolic diameter—mm	47 (6)	47 (6)	0.0 (− 3.1 to 2.5)	0.807
Left ventricular end-systolic diameter—mm	29 (6)	27 (5)	− 2.0 (− 1.2 to 3.8)	0.299
Interventricular septum—mm	9.1 (2.0)	9.2 (1.4)	− 0.1 (− 1.1 to 0.6)	0.612
Posterior wall—mm	9.0 (2.0)	9.2 (1.4)	− 0.2 (− 1.0 to 0.7)	0.723
Left ventricular mass index—g/m^2.7^	34.5 (10.9)	35.2 (10.2)	− 0.7 (− 5.5 to 4.1)	0.775
Relative wall thickness—%	0.385 (0.105)	0.391 (0.078)	− 0.006 (− 0.048 to 0.039)	0.829
Left ventricular ejection fraction—%	68 (10)	69 (8)	− 2 (− 6 to 3)	0.409
Left ventricular fractional shortening—%	38.5 (7.2)	41.1 (6.3)	− 2.6 (− 6.0 to 0.8)	0.128
Mitral annular plane systolic excursion—mm	13.0 (2.5)	12.5 (1.9)	0.5 (− 0.6 to 1.6)	0.377
Mitral annular peak systolic velocity *S*’—cm/s^a^	9.2 (2.0)	9.5 (1.7)	− 0.3 (− 1.1 to 0.5)	0.479
Peak *E* wave velocity—cm/s	76 (21)	70 (16)	6.0 (− 2.4 to 15.3)	0.153
* E*/*A* ratio^b^	1.29 (0.58)	1.14 (0.41)	0.16 (− 0.10 to 0.41)	0.223
*E* wave deceleration time—ms	207 (56)	210 (47)	− 3 (− 27 to 21)	0.809
Mitral annular *e*’ velocity—cm/s^a^	11.4 (3.3)	10.3 (2.1)	1.1 (− 0.3 to 2.5)	0.123
Mitral annular *e*’/*a*’ ratio^a^	1.13 (0.56)	1.00 (0.40)	0.13 (− 0.11 to 0.36)	0.279
Mitral *E*/*e*’ ratio^a^	6.63 (2.25)	7.22 (2.21)	− 0.59 (− 1.61 to 0.42)	0.252
Right heart				
Right atrial volume—ml	42 (21)	42 (14)	− 0 (− 9 to 9)	0.930
Right ventricular diastolic area—cm^2^	21.5 (5.1)	22.5 (4.7)	− 1.0 (− 3.1 to 1.3)	0.410
Right ventricular systolic area—cm^2^	13.1 (4.0)	13.6 (3.6)	− 0.5 (− 2.2 to 1.3)	0.586
Right ventricular fractional area change—%	0.389 (0.123)	0.391 (0.134)	− 0.002 (− 0.058 to 0.054)	0.939
Tricuspid annular plane systolic excursion—mm	21.7 (3.6)	20.6 (5.0)	1.1 (− 0.7 to 3.0)	0.219
Tricuspid annular peak systolic velocity *S*’—cm/s^a^	13.8 (3.0)	13.7 (2.9)	0.1 (− 1.3 to 1.3)	0.973
Tricuspid regurgitation peak velocity—m/s^c^	2.37 (0.40)	2.18 (0.48)	0.19 (− 0.04 to 0.42)	0.109
Tricuspid annular *e*’ velocity—cm/s^a^	14.6 (3.7)	13.3 (3.5)	1.3 (− 0.3 to 3.0)	0.100
Tricuspid annular *e*’/*a*’ ratio^a^	1.09 (0.59)	0.90 (0.37)	0.20 (− 0.06 to 0.44)	0.127
Systolic pulmonary artery pressure—mm Hg^c^	23 (7)	21 (9)	2 (− 1 to 8)	0.120
Lung ultrasound				
Score	1.0 (1.7)	3.7 (3.6)	− 2.7 (− 3.8 to − 1.8)	< 0.001

Values are means (SD). The 95% confidence intervals (CI) have not been adjusted for multiple testing and should not be used to infer definitive effects

^a^Tissue-Doppler imaging variables. *S*’, peak systolic velocities were measured either at the mitral or tricuspid annulus with the Doppler beam directed parallel to the myocardial walls; *e*’, early-diastolic myocardial velocity was measured either at the mitral or tricuspid annulus; *a*’, late-diastolic myocardial velocity was measured either at the mitral or tricuspid annulus

^b^*E*, early-wave trans-mitral peak velocity; *A*, late-wave trans-mitral peak velocity

^c^Measurement of tricuspid regurgitation peak velocity and calculation of systolic arterial pulmonary pressure could be performed in 84.6% of patients with mild-to-moderate COVID-19 and 85.2% of patients with severe COVID-19, and the mean value was imputed in the remaining patients

## Discussion

This study has involved patients who were hospitalized with COVID-19 in a University Hospital in North-East Italy and had been consecutively recruited, after hospital discharge, in a short-term follow-up. In these patients, cardiac structure and function that were assessed by two-dimensional, Doppler, and TDI echocardiography did not differ from controls who were matched for age, sex, body mass index, blood pressure, and major coexisting conditions. Moreover, structural and functional characteristics of the heart were comparable in patients who had recovered from mild-to-moderate or severe COVID-19. Despite the descriptive nature of this study due to its observational design, these echocardiographic findings together with normality of serum troponin, strongly suggest that patients who recover from COVID-19 do not have considerable pathological sequelae in the heart.

There is now considerable interest in the identification of persistent organ damage in survivors of COVID-19. This topic has ended up in the spotlight after demonstration of a pathological involvement of many organs different from the lung during the acute phase of infection [3]. Cardiac injury has been identified by detection of increased markers of myocardial damage during the critical stage of COVID-19 [17] and has been associated with an increased risk of death [6, 7]. Anecdotal necropsy studies conducted in subjects who died with COVID-19 have identified myocardial infiltration by interstitial mononuclear inflammatory cells, suggesting existence of a SARS-CoV-2-related myocarditis [18, 19]. Also, isolated cases of severe myocarditis with biventricular dysfunction have been reported in patients with COVID-19 [20, 21], even in the absence of overt pulmonary disease [22]. Although position statements of scientific societies have suggested use of targeted echocardiography for cardiac screening of patients with COVID-19 [13, 23], information obtained in the acute phase of illness is limited to a few studies. Right ventricular dilatation and reduced right ventricular fractional area change were observed in 27% of 74 patients with COVID-19 pneumonia and increased troponin levels, suggesting right ventricular systolic dysfunction as a possible consequence of acute pulmonary arterial hypertension [24]. Signs of pulmonary hypertension were observed with use of cardiac ultrasound in another investigation of 112 COVID-19 patients [25]. In a more recent systematic echocardiographic study conducted within 24 h of hospital admission in 100 COVID-19 patients, right ventricular dilatation and dysfunction have been reported in 39% of patients, left ventricular diastolic dysfunction in 16%, and left ventricular systolic dysfunction in 10% [26]. Thus, evidence obtained in the acute phase of COVID-19 has consistently and prevalently indicated a functional involvement of the right ventricle in a substantial proportion of patients. In this context, right ventricular dysfunction could be due to a hemodynamic overload caused by an increased pulmonary arterial pressure that, in turn, results from pulmonary thromboembolism.

Pulmonary thromboembolism has been demonstrated in patients who died with COVID-19 pneumonia as a possible consequence of a COVID-19 induced prothrombotic state [27]. The presence of a prothrombotic state in COVID-19 patients has also been supported by frequent detection of markedly increased D-dimer levels [28], and therefore, specific attention should be directed to assessment of right ventricular function even after recovery from infection. In this study, both right ventricular function and systolic pulmonary arterial pressure did not differ in patients who recovered from COVID-19 and controls. Also and most important, no significant abnormalities in right ventricular function and systolic pulmonary arterial pressure were found despite detection of persistent pulmonary involvement in COVID-19 patients who had been hospitalized with severe illness. These observations strongly suggest that cardiac changes that are associated with pulmonary involvement of COVID-19 during the acute phase of infection are rapidly reversed after recovery from disease.

More recently, two cross-sectional magnetic resonance studies have examined the heart of COVID-19 survivors in the early recovery phase. Puntmann et al. reported on 100 COVID patients who recovered from infection and had a cardiac magnetic resonance scan a mean of 71 days after diagnosis [29]. These subjects had larger left ventricular volumes and lower ejection fraction compared to controls, and 60% had evidence of ongoing myocardial inflammation. Another study by Rajpal et al. reported on 26 young athletes who recovered from COVID-19 and had a cardiac magnetic resonance from 11 to 53 days after diagnosis [30]. Of these patients, four had imaging data suggestive of myocarditis and eight of possible prior myocardial injury. Both these studies reported changes of the left ventricle, whereas, as indicated above, most magnetic resonance data obtained during the acute phase of COVID-19 reported involvement of the right ventricle. To notice, more than two-thirds of patients included in Puntmann’s paper were not hospitalized during the acute phase of COVID-19 and patients included in Rajpal’ s study were asymptomatic or mildly symptomatic, which suggests that their clinical conditions at presentation were not severe. This observation raises an important question on what could be the meaning, in terms of clinical relevance, of cardiac changes that were detected by magnetic resonance imaging in these patients. It would be definitely difficult to reconcile all these findings, because cardiac changes in people who recover from COVID-19 appear to be highly variable and this might depend on the technique that is used, the characteristics of patients in terms of severity of disease, and timing of examination. In particular, cardiac findings might differ significantly from the acute phase of infection and the short-term and long-term recovery phase and this is why it will be important to monitor the cardiac conditions of COVID-19 survivors even in a long-term follow-up.

Because cardiac abnormalities may persist in COVID-19 patients after viral clearance is achieved, or even arise in the chronic phase of disease, evaluation of cardiac structure and function in patients who recover from clinically relevant illness is of great importance. The present study was conducted during the pandemic peak in a geographic area of northeast Italy that has faced a slightly lower burden of infectivity, morbidity, and mortality than other regions in this country. However, during the study period mortality among COVID-19 patients who were admitted to our hospital with severe illness was 55%. Notably, the characteristics of patients included in our analysis overlap those that have been reported in much larger case series of patients with COVID-19 [31]. Consistent with previous descriptions of disease, our patients with severe COVID-19 were more frequently males, had lower lymphocyte count, and higher serum values of C-reactive protein, lactate dehydrogenase, procalcitonin, D-dimer, and interleukin 6 than patients with mild-to-moderate COVID-19 [2]. Therefore, despite the important limitation due to the small size of our study, these findings could be reasonably generalized to all subjects that recover from COVID-19.

Additional limitations of our study should be highlighted. First, lack of echocardiographic measurements obtained during the hospital stay in the acute phase of COVID-19 does not allow any longitudinal assessment of cardiac changes. Second, inclusion of consecutive COVID-19 patients who attended the follow-up visits might have led to a selection bias that occurs almost inevitably in observational studies. Third, although the matching process of control subjects was accurate for demographic and clinical variables, uncontrolled confounders might have affected the final results. For instance, significant differences in the frequency of use of antivirals and interleukin-6 antagonists between patients with mild-to-moderate and severe COVID-19 could have affected the cardiac outcome. Last, because data were collected after a median of 6 weeks after COVID-19 diagnosis, these do not provide information on the cardiac outcome of infection in a longer time span.

In conclusion, in this study we did not identify structural or functional abnormalities in the heart of survivors of COVID-19 more than a month after the first detection of infection. No abnormalities were also observed in the heart of patients who recovered from severe COVID-19. These findings suggest that patients who recover from COVID-19 do not have considerable cardiac sequelae, but this evidence needs to be further investigated in larger and longer-term studies.

## Supplementary Information

Below is the link to the electronic supplementary material.

Supplementary file1 (DOCX 30 KB)
