# Supplementary material for: Short-term cardiac outcome in survivors of COVID-19: a systematic study after hospital discharge
Source: Clin Res Cardiol. 2021 Jan 22;110(7):1063–72. doi: 10.1007/s00392-020-01800-z (PMC7820534; doi:10.1007/s00392-020-01800-z)
Supplement: Supplementary file 1 — Supplementary file1 (DOCX 30 KB) [file 392_2020_1800_MOESM1_ESM.docx]

**Supplementary Material**

**Short-Term Cardiac Outcome in Survivors of COVID-19:**

**a Systematic Study After Hospital Discharge**

Leonardo A. Sechi^1^, M.D., Gianluca Colussi^2^, M.D., Luca Bulfone^1^, M.D., Gabriele Brosolo^2^, M.D., Andrea Da Porto^1^, M.D., Maddalena Peghin^3^, M.D., Vincenzo Patruno^4^, M.D., Carlo Tascini^3^, M.D.,

Cristiana Catena^2^, M.D., Ph.D.

From the University of Udine, Department of Medicine, ^1^Division of Internal Medicine (L.A.S., L.B., A.D.P.), ^2^Cardiovascular Unit (G.C., G.B., C.C.), ^3^Division of Infectious Diseases (M.P., C.T.),

and ^4^Division of Pneumology (V.P.), 33100 Udine, Italy

**Supplementary Methods**

**Echocardiography**

We performed two-dimensional, Doppler, and tissue-Doppler imaging (TDI) transthoracic cardiac ultrasound examination in survivors of COVID-19 after hospital discharge. Cardiac ultrasound examination was performed with use of appropriate personal protective equipment [1] by experienced investigators (C.C. and G.C.) who were blinded to the clinical and laboratory data, in accordance with the joint recommendations of the American Society of Echocardiography and the European Association of Cardiovascular Imaging [2], as described previously [3]. Measurements were obtained with the patient in the partial left decubitus position, with a commercial machine (Aplio CV, Toshiba Medical System, Tokyo, Japan) and a 2.5 MHz transducer, under two-dimensional cross-sectional control and simultaneous electrocardiographic tracing.

Left and right atrial diameters, areas, and volumes were measured by an apical four-chamber view at the end of left ventricular systole by tracing the border of the atria and excluding the confluences of the pulmonary and cava veins and the left atrial appendage. The atrioventricular interface was considered as the mitral and tricuspid valve planes.^5^

Left ventricular cavity size including linear internal dimensions were taken at end-diastole and end-systole, were indexed to the body surface area and were used to calculate parameters of left ventricular function. Linear internal measurements of the left ventricle and its wall thickness were obtained in the parasternal long-axis view, perpendicularly to the left ventricular long-axis and measured immediately below the level of the mitral valve leaflet tips with a two-dimensional-guided M-mode approach. Left ventricular mass index was calculated by the Penn Convention formula and normalized for body height, being expressed as grams/m^2.7^. The relative wall thickness was used for assessment of left ventricular geometry [2].

Left ventricular systolic function was assessed by measurement of ejection fraction, left ventricular fractional shortening, TDI-derived mitral annular peak systolic velocity (S’), and the mitral annular plane systolic excursion (MAPSE) [4,5]. Left ventricular diastolic function was evaluated both with conventional pulsed Doppler trans-mitral flow and TDI in all patients. Conventional pulsed Doppler recordings were obtained at the level of the mitral valve tips at late expiration. Early- (E) and late-wave (A) trans-mitral diastolic peak velocities, the E/A ratio, and E wave deceleration time were measured. For TDI, a 3.5-mm sample volume was used and the Doppler beam was directed parallel to the myocardial walls. Early-diastolic and late-diastolic velocities of septal and lateral myocardial portions were measured at the level of mitral valve annulus, and the mean values (*e’* and *a’*, respectively) were calculated on 5 consecutive cardiac cycles at end-expiration, together with the average *e’/a’* and E/*e’* ratios [6].

Right ventricular dimensions were measured from a right ventricle-focused apical four-chamber view obtained with either lateral or medial transducer orientation, to obtain the image with the left ventricular apex at the center of the scanning sector, while displaying the largest basal right ventricular diameter. With this method we obtained measurements of diastolic and systolic areas, and basal, mid, and longitudinal diameters [7].

Right ventricular systolic function was assessed according to current guidelines by the tricuspid annular plane systolic excursion (TAPSE) that was obtained from the tricuspid lateral annulus, the two-dimensional right ventricular ejection fraction (fractional area change) expressed as percentage after making sure that the entire right ventricle was in the view, including the apex and the lateral wall in both systole and diastole, and the TDI-derived tricuspid lateral annular systolic velocity (S’).^7^ Right ventricular diastolic function was evaluated by TDI in all patients. Early-diastolic and late-diastolic velocities were measured at the level of tricuspid valve annulus, and the mean values (*e’* and *a’*, respectively) were calculated on 5 consecutive cardiac cycles at end-expiration, together with the average *e’/a’* ratio [7].

Systolic pulmonary artery pressure was calculated in patients in whom tricuspid regurgitation could be detected by color Doppler, with measurement of tricuspid regurgitation peak velocity (TRPV) at the tricuspid plane. The following formula was used for calculation: (TRPV^2^ x 4) + atrial pressure (estimated from the inferior vena cava diameter) [6].

**References**

1. Kirkpatrick JN, Mitchell C, Taub C, Kort S, Hung J, Swaminathan M (2020) ASE statement on protection of patients and echocardiography service providers during the 2019 novel coronavirus outbreak: endorsed by the American College of Cardiology. J Am Soc Echocardiogr 33:648-653. doi:10.1016/j.jacc.2020.04.002

2. Lang RM, Badano LP, Mor-Avi V, Afilalo J, Armstrong A, Ernande L, et al (2015) Recommendations for cardiac chamber quantification by echocardiography in adults: an update from the American Society of Echocardiography and the European Association of Cardiovascular Imaging. J Am Soc Echocardiograph 28:1-39. doi:10.1016/j.echo.2014.10.003

3. Catena C, Verheyen N, Pilz S, Kraigher-Krainer E, Tomaschitz A, Sechi LA, et al (2015) Plasma aldosterone and left ventricular diastolic function in treatment-naive patients with hypertension: tissue-Doppler imaging study. Hypertension 65:1231-1237. doi:10.1161/HYPERTENSIONAHA.115.05285

4. Carlsson M, Ugander M, Mosen H, Buhre T, Arheden H (2007) Atrioventricular plane displacement is the major contributor to left ventricular pumping in healthy adults, athletes, and patients with dilated cardiomyopathy. Am J Physiol Heart Physiol 292:H1452-H1459. doi: 10.1152/ajpheart.01148.2006

5. Ommen SR, Nishimura RA, Appleton CP, Miller FA, Oh JK, Redfield MM, et al (2000) Clinical utility of Doppler echocardiography and Tissue Doppler Imaging in the estimation of left ventricular filing pressures: a comparative simultaneous Doppler catheterization study. Circulation 102:1788-1794. doi: 10.1161/01.cir.102.15.1788

6. Nagueh SF, Smiseth OA, Appleton CP, Byrd BF, Dokainish H, Edvardsen T et al (2016) Recommendations for the evaluation of left ventricular diastolic function by echocardiography: an update from the American Society of Echocardiography and the European Association of Cardiovascular Imaging. J Am Soc Echocardiogr 29:277-314. doi:10.1016/j.echo.2016.01.011

7. Rudski LG, Lai WW, Afilalo J, Hua L, Handschumaker MD, Chandrasekaran K, et al (2010) Guidelines for the echocardiographic assessment of the right heart in adults: a report from the American Society of Echocardiography endorsed by the European Association of Echocardiography, a registered branch of the European Society of Cardiology, and the Canadian Society of Echocardiography. J Am Soc Echocardiogr 23:685-713. doi:10.1016/j.echo.2010.05.010

**Supplementary Table 1.** Clinical Score for Severity of Illness in Patients with COVID-19

| Level 1 | Fever (≥37.5°C), with or without mild additional signs and symptoms, with arterial oxygen saturation ≥94% at rest while breathing in ambient air and during a walking test, without any lung infiltrates on chest X-ray |
| --- | --- |
| Level 2 | Fever, signs of a pulmonary involvement on chest X-ray or CT-scan, arterial oxygen saturation ≥94% and/or arterial partial oxygen pressure >60 mm Hg |
| Level 3 | Fever and overt respiratory failure (respiratory rate ≥30 breaths per minute; arterial oxygen saturation ≤93% and/or arterial partial oxygen pressure <60 mm Hg; ratio of the partial pressure of arterial oxygen to the fraction of inspired oxygen [Pao_2_:FIo_2_] <300) |
| Level 4 | Overt respiratory failure and suspected initial acute respiratory distress syndrome (ARDS) |
| Level 5 | Confirmed ARDS on CT scan with severe hypoxemia and evidence of bilateral lung infiltrates and pulmonary edema |

**Supplementary Table 2.** Laboratory Variables of COVID-19 Patients During Hospitalization and at Follow-up.*

| **Variable** | **Study Patients**  **Hospital**  **(N=105)** | **Study Patients**  **Follow-up**  **(N=105)** | **P-value** |
| --- | --- | --- | --- |
| Hemoglobin – g/dl | 14.0 (1.2) | 13.8 (1.3) | 0.127 |
| White-cell count – per mm^3^ | 5,992 (2,997) | 6,414 (2,659) | 0.307 |
| Lymphocyte count – per mm^3^ | 948 (410) | 1,672 (698) | <0.001 |
| Platelet count – per mm^3^ | 203,000 (76,000) | 245,000 (77,000) | <0.001 |
| Glucose – mmol/liter | 6.18 (1.78) | 5.94 (1.67) | 0.738 |
| Creatinine – μmol/liter | 86.6 (24.7) | 82.0 (22.8) | 0.185 |
| Sodium – mmol/liter | 139 (3) | 140 (2) | 0.109 |
| Potassium – mmol/liter | 3.99 (0.45) | 4.11 (0.37) | <0.001 |
| Alanine aminotransferase – U/liter | 21 [15-32] | 19 [17-25] | 0.328 |
| Aspartate aminotransferase – U/liter | 26 [21-36] | 24 [17-32] | 0.065 |
| Total bilirubin – μmol/liter | 8.2 [6.6-11.1] | 7.9 [5.8-10.3] | 0.141 |
| Creatine kinase – U/liter | 88 [60-140] | 64 [48-98] | <0.001 |
| Lactate dehydrogenase – U/liter | 465 [374-687] | 420 [355-497] | <0.001 |
| C-reactive protein – mg/liter | 52.8 [16.8-103.2] | 1.7 [0.7-6.8] | <0.001 |
| Procalcitonin – ng/ml | 0.06 [0.03-0.14] | 0.02 [0.01-0.03] | <0.001 |
| D-dimer – ng/ml | 555 [331-1087] | 342 [226-655] | <0.001 |

*Normally distributed values are shown as means (SD). Variables with skewed distribution are shown as medians [interquartile range]. To convert the values for glucose to milligrams per deciliter, divide by 0,0555; to convert the values for creatinine to milligrams per deciliter, divide by 88.434; to convert the values for total bilirubin to milligrams per deciliter, divide by 17.104.

**Supplementary Table 3.** Clinical Characteristics and Coexisting Conditions of Survivors of COVID-19 and Matched Controls.*

| **Characteristic** | **Study Patients**  **(N=105)** | **Controls**  **(N=105)** |
| --- | --- | --- |
| Age – yr | 57 (14) | 57 (14) |
| Female sex – no. (%) | 49 (47) | 49 (47) |
| Body mass index | 26.22 (4.76) | 26.20 (4.18) |
| Systolic blood pressure – mm Hg | 132 (15) | 133 (18) |
| Diastolic blood pressure – mm Hg | 79 (10) | 80 (12) |
| Current smokers – no. (%) | 16 (15) | 17 (16) |
| Coexisting conditions – no. (%) | | |
| Hypertension | 31 (30) | 31 (30) |
| Diabetes mellitus | 8 (8) | 7 (7) |
| Hyperlipidemia | 9 (9) | 10 (10) |
| Coronary artery disease | 8 (8) | 8 (8) |
| Congestive heart failure | 2 (2) | 2 (2) |
| Atrial fibrillation | 9 (9) | 8 (7) |
| Chronic obstructive pulmonary disease | 3 (3) | 4 (4) |

*Values are means (SD). Cases of Covid-19 were diagnosed between March 15 and April 15, 2020. Controls were selected by use of a nearest neighbor matching strategy from a large internal database of subjects that are representative of the general population and were exactly matched for age, sex, and hypertension, whereas propensity score models were used to match for body mass index, smoking, diabetes mellitus, hyperlipidemia, coronary artery disease, congestive heart failure, atrial fibrillation, and chronic obstructive pulmonary disease.

**Supplementary Table 4.** Drugs Used During Hospitalization for Treatment of COVID-19 in Patients with Mild-Moderate and Severe Disease.*

| **Drug** | **Mild-Moderate**  **COVID-19**  **(N=78)** | **Severe**  **COVID-19**  **(N=27)** |
| --- | --- | --- |
| **Antimalarials no. (%)** | | |
| Hydroxychloroquine | 51 (63) | 26 (96) |
| **Antivirals no. (%)** | | |
| Darunavir cobicistat | 25 (32) | 17 (63) |
| Lopinavir/Ritonavir | 22 (28) | 15 (56) |
| Oseltamivir | 1 (1) | 1 (4) |
| **Antibiotics no. (%)** | | |
| Amikacin | 0 (0) | 1 (4) |
| Amoxicillin clavulanate | 2 (3) | 0 (0) |
| Azithromicin | 30 (39) | 10 (37) |
| Ceftaroline | 0 (0) | 2 (7) |
| Ceftriaxone | 4 (5) | 2 (7) |
| Ceftobiprole | 0 (0) | 1 (4) |
| Ceftolozane tazobactam | 0 (0) | 1 (4) |
| Levofloxacine | 1 (1) | 0 (0) |
| Linezolid | 0 (0) | 1 (4) |
| Meropenem | 0 (0) | 2 (7) |
| Piperacillin tazobactam | 1 (1) | 4 (15) |
| **Steroids no. (%)** | | |
| Dexamethasone | 2 (3) | 3 (11) |
| Methylprednisolone | 1 (1) | 4 (15) |
| **Miscellaneous no. (%)** | | |
| Amiodarone | 2 (3) | 3 (11) |
| Anakinra | 0 (0) | 1 (4) |
| Enoxaparin | 16 (21) | 12 (44) |
| Icatibant | 0 (0) | 2 (7) |
| High-dose immunoglobulins | 0 (0) | 3 (11) |
| Tocilizumab | 5 (6) | 15 (56) |

*Data were collected from drug charts of patients admitted to the Hospital. Indication for use of tocilizumab was detection of increased serum levels of interleukin-6.
